# New Insight into Benign Tumours of Major Salivary Glands by Proteomic Approach

**DOI:** 10.1371/journal.pone.0071874

**Published:** 2013-08-26

**Authors:** Elena Donadio, Laura Giusti, Veronica Seccia, Federica Ciregia, Ylenia da Valle, Iacopo Dallan, Tiziana Ventroni, Gino Giannaccini, Stefano Sellari-Franceschini, Antonio Lucacchini

**Affiliations:** 1 Department of Pharmacy, University of Pisa, Pisa, Italy; 2 1st Ear Nose Throat Unit, Azienda Ospedaliero Universitaria Pisana, Pisa, Italy; Ospedale Pediatrico Bambino Gesu', Italy

## Abstract

Major salivary gland tumours are uncommon neoplasms of the head and neck. The increase of precise pre-operative diagnosis is crucial for their correct management and the identification of molecular markers would surely improve the required accuracy. In this study we performed a comparative proteomic analysis of fine needle aspiration fluids of the most frequent benign neoplasms of major salivary glands, namely pleomorphic adenoma and Warthin's tumour, in order to draw their proteomic profiles and to point out their significant features. Thirty-five patients submitted to parotidectomy were included in the study, 22 were identified to have pleomorphic adenoma and 14 Warthin's tumour. Fine needle aspiration samples were processed using a two-dimensional electrophoresis/mass spectrometry-based approach. A total of 26 differentially expressed proteins were identified. Ingenuity software was used to search the biological processes to which these proteins belong and to construct potential networks. Intriguingly, all Warthin's tumour up-regulated proteins such as Ig gamma-1 chain C region, Ig kappa chain C region and Ig alpha-1 chain C region and S100A9 were correlated to immunological and inflammatory diseases, while pleomorphic adenomas such as annexin A1, annexin A4, macrophage-capping protein, apolipoprotein E and alpha crystalline B chain were associated with cell death, apoptosis and tumorigenesis, showing different features of two benign tumours. Overall, our results shed new light on the potential usefulness of a proteomic approach to study parotid tumours and in particular up regulated proteins are able to discriminate two types of benign parotid lesions.

## Introduction

Major salivary gland tumours (MSGT) are uncommon neoplasms of the head and neck corresponding to about 8% of the cancers of the head and neck, and they can be malignant or benign. Malignant MSGT account for about 7% of epithelial cancers of the head and neck in the United States, with an annual incidence of about 1 per 100,000 population and a similar incidence is reported in Europe. Benign MSGT occur more frequently, but the precise incidence is difficult to quantify as they are obviously not included in cancer registry data [1].

The management of MSGT is based on clinical assessment, radiologic investigation, and cytologic study of fine needle aspiration biopsy (FNAB) specimens. FNAB is a relatively painless procedure for rapid diagnosis, as the lesions are readily accessible [2]. Nevertheless, since different studies indicate higher values of specificity than sensitivity, parotid carcinomas are frequently misdiagnosed as a benign process (false-negative) than benign lesions are diagnosed as carcinomas (false-positive). A recent retrospective study indicates that among the false-negative results, 5 out of 11 cases of the cytological misdiagnosis were Warthin's tumours (WT), while among the false-positive results WT, pleomorphic adenomas (PA) and lymphoepithelial lesions were the most common histological types [3]. Tryggvason and co-authors [4] reported diagnosis of 1,128 salivary gland FNAB. These diagnoses contained very few false-positives, while false-negatives were more common especially for carcinoma ex-PA which was interpreted as PA for all 6 cases of misdiagnosis.

Nagel and collaborators [5] underlined that PA is most frequently confused in cytology with adenoid cystic carcinomas, principally because adenoid cystic carcinomas and PA are composed of the same cellular and extracellular elements. Diagnosis of a pleomorphic carcinoma may be confused with a mucoepidermoid carcinoma according to Stewart [6].

In cases of WT with predominance of the lymphoid component, squamous cells and necrotic/mucoid debris, there can be a significant diagnostic challenge because the squamous element, in particular, is common to squamous cell carcinomas and mucoepidermoid carcinomas [7]. Aspirates dominated by lymphoid components can be indistinguishable from a reactive lymph node or sialadenitis. WT can be misdiagnosed with lymphocyte-rich acinic cell carcinoma [8].

The increase of the pre-operative diagnosis is crucial for the correct management of such neoplasms, and, in that sense, the identification of molecular markers would surely improve the required accuracy. A more reliable diagnostic tool would help distinguish parotid neoplasms from parotid lymphomas, avoiding unnecessary surgery.

The application of proteomic analysis to different biological fluids has disclosed significant differences in concentration of various proteins between normal and affected subjects, opening the door to the identification of potential biomarkers useful in the early diagnosis and treatment of the disease. At this time, proteomic application in head and neck cancer are confined to search potential markers in tissue [9], [10], blood [11] and saliva samples [12] but, to our knowledge, no studies are reported about the application of proteomic analysis to fine needle aspiration (FNA) fluid of the parotid tumour. Proteome analysis has been shown to be a powerful tool for studying human diseases and for identifying novel prognostic, diagnostic, and therapeutic markers. This approach has been successfully used to determine proteomic profile of human thyroid FNA [13] and successively to identify thyroid cancer biomarkers [14].

In this paper, using a combination of two-dimensional electrophoresis (2-DE) and mass spectrometry (MS), the proteome profile of FNA samples of WT parotid tumours with those of PA samples was compared. The aim of our study is to explore the possibility of performing a proteomic analysis on the most frequent benign neoplasms of major salivary glands, namely PA and WT, to draw their proteomic profile and to point out their most significant features in order to improve FNA cytology accuracy in the pre-operative assessment of such lesions. Differential expression of proteins of interest has been validated by Western Blot (WB) analysis.

## Materials and Methods

### Materials

Iodoacetamide, dithiothreitol (DTT), 3-[(3-cholamidopropyl) dimethylammonio]-1-propanesulfonate (CHAPS), urea, thiourea, glycerol, sodium dodecyl sulfate (SDS), tetramethylethylenediamine (TEMED), ammonium persulfate, glycine and 30% acrylamide-N,N,N bisacrylamide were acquired from Applichem (Germany). IPGs pH 3–10 NL, pharmalyte 3–10 and dry strip cover fluid were purchased from GE Health Care Europe (Uppsala, Sweden). Enhanced chemiluminescence (ECL) detection system was purchased from PerkinElmer (MA, USA). Anti-Selenium binding protein 1 (SBP1) was purchased from Sigma-Aldrich (MO, USA); anti-protein S100-A9 was purchased from Santa Cruz Biotechnology (CA, USA); anti-macrophage-capping protein (CAPG) and anti-S-formylglutathione hydrolase (ESD) were purchased from Novus Biologicals (CO, USA); anti-glyceraldehyde-3-phosphate dehydrogenase (GAPDH), anti-Annexin A1 (ANXA1), anti-alpha crystalline B chain (CRYAB) and anti-superoxide dismutase [Cu-Zn] (SOD1) were purchased from Cell Signaling Technology, Inc. (MA, USA); anti-Ig gamma-1 chain C region (IGHG1) was purchased from Abnova (Taiwan). Secondary antibodies (horseradish peroxidase (HRP)-conjugated) were purchased from Stressgen (Belgium) (donkey anti-rabbit) and from Santa Cruz Biotechnology (donkey anti-goat). All other reagents were acquired from standard commercial sources and were of the highest grade available.

### Patients

Thirty-five patients submitted to parotidectomy were included in the study (Table 1). Diagnostic results were obtained from pathology reports that accompanied each specimen and were confirmed after histological examination. The clinical profile and tumour classification are summarised in Table 1.

**Table 1:** Patients' demographics and clinical features.

	No Patients	Gender F/M	No Lesions	No Parotid Gland (%)	No Submandibular gland (%)	Dimensions (mm; mean)	Range (mm; min-max)
**Total**	36	15/11	37	35 (94.6)	2 (5.4)	25	9–50
**WT**	14	4/10	15	15 (100)	0 (0)	25	17–40
**PA**	22	11/11	22	20 (90.9)	2 (9.1)	24	9–50

WT: Warthin's Tumor; PA: Pleomorphic Adenoma.

**Figure 1:**
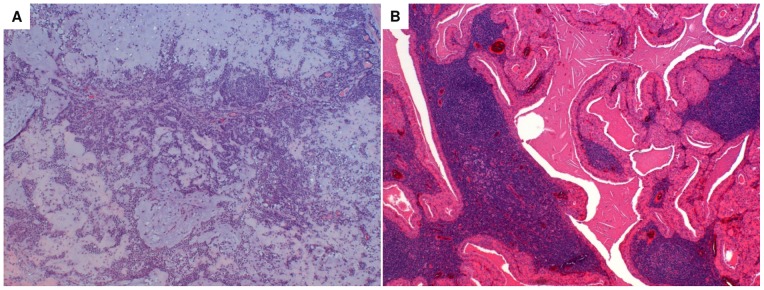
Typical hystological image of PA and WT. The essential components of PA are the capsule, epithelial and myoepithelial cells and mesenchimal or stromal elements. The epithelial component consists of a wide variety of cytologically bland cells, forming sheets or duct-like structures. Ducts show a luminal layer of cuboidal cells and an abluminal layer of myoepithelial cells. The stromal mesenchimal-like component may be myxoid, mucoid, cartilagineous or hyalinised (Haematoxylin & Eosin staining, magnification 4X) (panel A). The tumour is sharply demarcated with a thin capsule, and is characterized by cystic and solid areas composed of lymphoid stroma and the epithelial component. The lymphoid stroma shows varying degrees of reactivity and, usually, germinal centers. The epithelial component comprises two cell layers: the oncocytic luminal cells, tall and columnar with palisading nuclei, and the flattened or cuboidal abluminal cells. Papillary structures usually project into the limina of cystic spaces (Haematoxylin & Eosin staining, magnification 4X) (panel B).

Among the 36 patients, 22 were identified to have PA and 14 WT, the typical hystological images are shown in Figure 1. The histopathological classification was performed in accordance with the World Health Organization classification [15].

### Ethics Statement

All patients gave their informed consent for proteomic studies. This study was approved by the Local Ethics Committee (Comitato per la sperimentazione clinica dei farmaci, Azienda Ospedaliera Universitaria Pisana), and signed consent forms were obtained for all patients.

### Sample collection and preparation

Immediately after surgical removal of the parotid, a FNA was made on the nodule. After passing the needle through the tissue 3 or 4 times, 1 mL of saline solution was aspirated with the same syringe. This fluid was immediately centrifuged at 2300 *g* for 10 min at 4 °C The supernatants were stored at −80°C until use.

FNA proteins were precipitated with 1% of Trichloroacetic acid containing 1 mM dithiothreitol (DTT). Protein pellets were washed extensively with acetone at 0°C. The resulting pellets were resuspended in rehydration solution (7 M Urea, 2 M thiourea, 4% CHAPS, 60 mM DTT, 0.002% bromophenol blue) and incubated for 30 min. at room temperature. After incubation, the samples were centrifuged for 10 min at 14,000 g to remove undissolved material. Protein concentrations were measured with a RC-DC Protein Assay from Bio-Rad using bovine serum albumin as the standard. All samples were stored at −80°C until analysis.

### 2-DE

Two pools belonging to the PA and WT samples were made and each pool was submitted to 2-DE in triplicate. Isoelectrofocusing (IEF) was carried out by using 18 cm Immobiline Dry-Strips with a non-linear, pH 3–10, gradient. Two-hundred and fifty μg of protein was filled to 350 μl of rehydration buffer supplemented with 1% (v/v) pharmalyte, pH 3–10. IEF was performed at 16°C on a Ettan IPGphor II apparatus (GE-Healthcare) as previously described [13], [16]. The second dimension was performed using the PROTEAN-II Multi Cell system (Bio-Rad, CA, USA) on 12% polyacrylamide gel (18 cm x 20 cm x 1.5 mm) at 96 mA for 1 hour and at 192 mA for 15 hours applying a continuous buffer system.

### Staining and image analysis

The gels were stained with Ruthenium II tris (bathophenanthroline disulfonate) tetrasodium salt (SunaTech Inc) according to Rabilloud and Lelong [17]. After electrophoresis, gels were fixed for 1 hr. in 1% phosphoric acid (v/v, starting from commercial 85% phosphoric acid) and 30% ethanol; then gels were stained overnight with 1 mM ruthenium complex (RuBP) in 1% phosphoric acid–30% ethanol, destained for 4–6 hrs. in 1% phosphoric acid–30% ethanol and rinsed in water (one rinse, 10 min) prior to imaging. The stained gels were acquired with on fluorescence by “ImageQuant LAS4010” (GE-Healthcare), and the images were analyzed with the Progenesis SameSpot (Non linear - Dynamics) software as previously described [13]. The software included statistical analysis calculations such as Anova p-value and False Discovery Rate (q-values). A comparison between PA and WT gels pool was performed. The protein spots with a >2-fold spot quantity change between the two pools, p<0.05 and q value <0.05 were selected and identified (herein referred to as ‘‘differentially expressed proteins’’).

### NanoLC-ESI-MS/MS analysis by LTQ-Orbitrap Velos Analysis

Spots of interest were cut out from gel reference and the nanoLC-ESI-MS/MS analysis by LTQ-Orbitrap Velos was performed as previously described [16]. Peak lists were generated from raw orbitrap data using the embedded software from the instrument vendor (extract_MSN.exe). The monoisotopic masses of the selected precursor ions were corrected using an in-house written Perl script. The peak list files were searched against the SwissProt/trEMBL database (Release 15.10 of 03-Nov-2009) using Mascot (Matrix Sciences, London, UK). Human taxonomy (98529 sequences) was specified for database searching. The parent ion tolerance was set to 10 ppm. Oxidation of methionine was specified in Mascot as a variable modification. Trypsin was selected as the enzyme, with one potential missed cleavage, and the normal cleavage mode was used. The mascot search was validated using Scaffold 3.4.9 (Proteome Software, Portland, OR). Only proteins matching with two different peptides with a minimum probability score of 95% were considered to be identified. The reference limit to p<0.05 for the probabilistic scores of MS/MS assignment was 45. When multiple proteins were identified in a single spot, the proteins with the highest number of peptides were considered as those corresponding to the spot.

### Western blot analysis

For western blotting (WB), all FNA samples were processed to validate different protein expressions found with 2DE analysis. Aliquots of each FNA sample were mixed with a SDS sample buffer (Laemmli solution). Amount of the samples **(**ranging from 10 to 60 μg of proteins) were run on 12% SDS-PAGE gels, and transferred onto nitrocellulose membranes (0.2 μm) using a voltage of 100 V for 30 min. (Mini vertical gel system, Biorad). Non-specific binding was prevented by blocking the membranes with 3% low fat dried milk, 0.2% (v/v) Tween 20 in PBS (10 mM NaH2PO4, pH 7.4, 0.9% NaCl) (PBS/milk/Tween) for 1 hr. at room temperature. After blocking, the membranes were incubated with an appropriately diluted primary antibody in a blocking buffer overnight at 4°C (1∶200 dilution for anti-protein S100-A9; 1∶500 dilution for anti-CAPG and anti-ESD; 1∶1000 dilution for anti-SBP1, anti-GAPDH, anti-ANXA1, anti-CRYAB and anti-SOD1; 1∶5000 dilution for anti-IGHG1). After four washes with PBS/milk/Tween, the immunocomplexes were detected using a peroxidase-labelled secondary antibody (donkey anti-rabbit 1∶10000 dilution, donkey anti-goat 1∶5000). Immunoblots were developed using the ECL detection system. The chemiluminescent images were acquired by LAS4010 (GE Health Care). For the comparison of protein expression levels between PA and WT samples, the antigen-specific bands were quantified using the Image Quant-L (GE Health Care).

### Statistical analysis

Statistical analysis of the two classes (PA and WA) of gels was performed by Progenesis Same Spot (Nonlinear Dynamics). The software included the following statistical analysis calculations: Anova p-value, False Discovery Rate (q-values). Statistical significant differences in the immunoreactive bands were calculated by the Student t test.

### Signalling pathway analysis

Functional pathway and network analyses were generated using the Ingenuity pathways analysis (IPA) software v14400082 (2012, Ingenuity Systems, www.ingenuity.com). IPA identified the pathways from the IPA library of canonical pathways that were most significant to the data set. Proteins that met the expression ratio with a cut-off of >2.0, a p value cut-off of 0.05 for differential expression, and that were associated with a canonical pathway in the Ingenuity Pathways Knowledge Base, were considered for the analysis. HUGO or Swiss-Prot accession numbers and official gene symbols were inserted into the software along with the corresponding comparison ratios between the groups. The network proteins associated with biological functions and/or diseases in the Ingenuity Pathways Knowledge Base were considered for the analysis. These networks were scored for the degree of relevance, with values >3 having a 99.9% confidence level of not being generated by random chance alone. The genetic networks that were created describe functional relationships between gene products based on known associations in the literature.

## Results

### Proteomic analysis of PA and WT FNA

**Figure 2:**
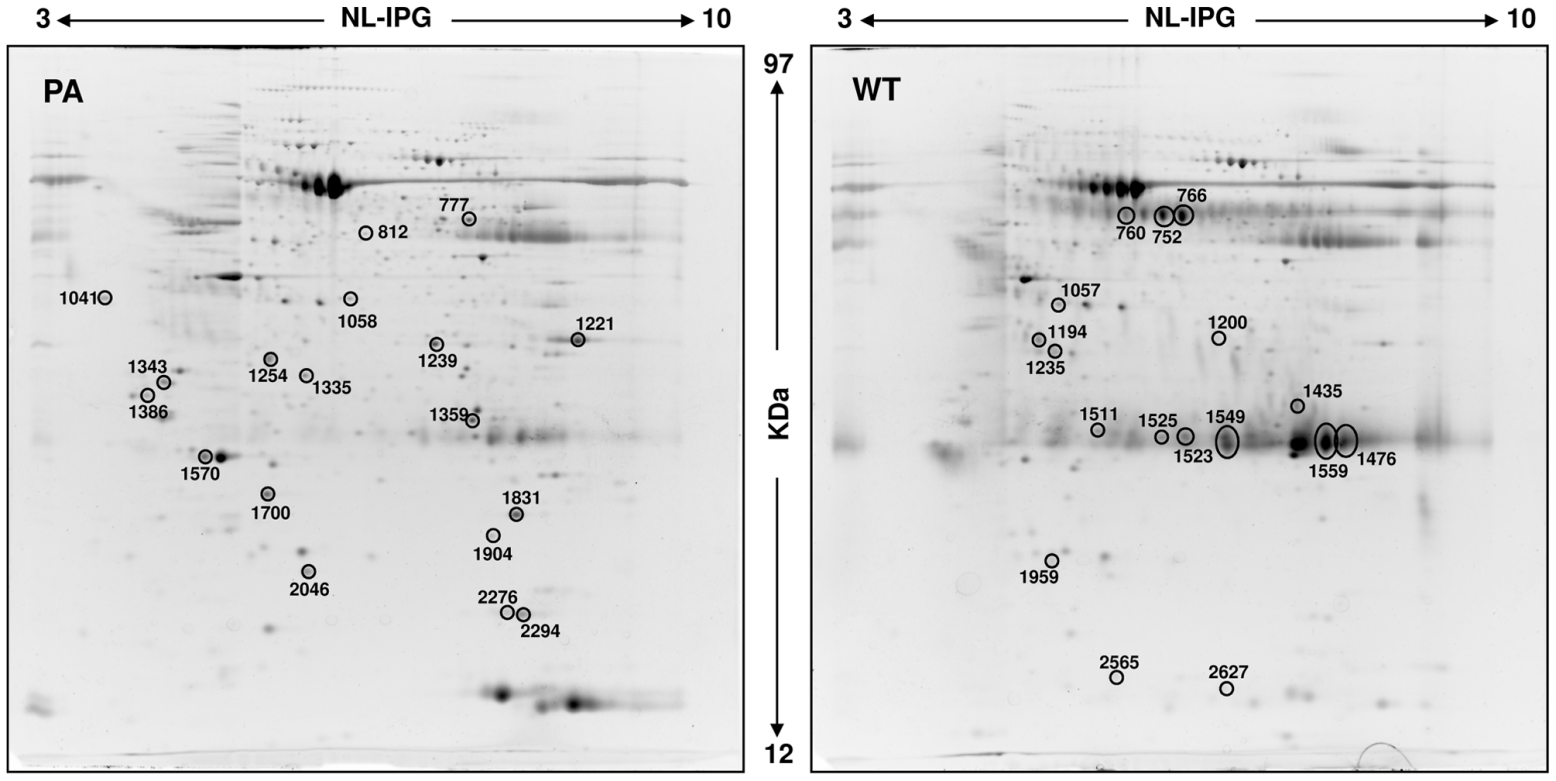
Representative 2D gel maps of PA and WT FNA pool samples. A total of 250 µg of proteins was separated by 2D using an 18 cm pH 3–10 NL strip and 12.5% SDS-PAGE. Proteins were detected by Ruthenium II tris (bathophenanthroline disulfonate) tetrasodium salt staining. The maps were analyzed by Progenesis Same Spot (Nonlinear Dynamics) software. Spots numbers indicate all the proteins identified by MS/MS and refer to the number reported in Table 2 (PA) and Table 3 (WT).

A comparative proteomic analysis was performed on PA and WT FNA samples using 2-DE followed by nanoLC-ESIMS/ MS. Typical 2D gel images of PA and WT FNA samples are shown in Fig. 2. By computational 2D gel image comparison, a total of 34 proteins differentially expressed were found with a fold variation major than 2. Differentially expressed protein spots were subsequently subjected to nano LC-ESI-MS/MS analysis and identified, leading to the identification of 26 different proteins. A list of identified proteins, MW, pI, score and coverage values of MS/MS, fold-change in expression levels and p values are shown in Tables 2 (PA) and 3 (WT). Interestingly, several spots (with pI and MW observed different from theoretical) for the same protein were found for serum albumin (n. 752, 760, 766, 1194), for fibrinogen beta chain (n. 777, 1057) suggesting the occurrence of post translational modification which often are associated with different pathological state as reported in type 1 diabetes [18].

**Table 2:** MS/MS data of protein spots up-regulated in PA FNA samples.

Spot no.	ID	Protein name	Gene name	Th	Obs	Matched peptides	Coverage(%)	Best ion score	Peptides identified	FV	p-value
				MW/pI	MW/pI						
**777**	P02675	Fibrinogen beta chain	FGB	56/ 8.5	51/ 5.8	12	27	75.8	(K)LESDVSAQmEYcR(T)	2.8	6.480e^−5^
**812**	Q13228	Selenium-binding protein 1	SELENBP1	52/5.9	48/5.6	11	29	94.3	(R)NTGTEAPDYLATVDVDPK(S)	3.6	0.002
**1041**	Q9UK22	F-box only protein 2	FBOXO2	33/4.3	39/3.9	3	12	87.3	(R)VLAALPAAELVQAcR(L)	4.1	1.936e^−5^
**1058**	P40121	Macrophage-capping protein	CAPG	38/5.8	39/5.6	5	26	59.6	(R)QAALQVAEGFISR(M)	3.0	8.584e^−5^
**1221**	P04406	Glyceraldehyde-3-phosphate dehydrogenase	GAPDH	36/8.6	35/6.9	10	36	76.4	(R)VPTANVSVVDLTcR(L)	2.6	0.016
**1239**	P04083	Annexin A1	ANXA1	39/6.6	34/5.8	18	45	102.5	(R)KGTDVNVFNTILTTR(S)	2.0	0.002
**1254**	P02649	Apolipoprotein E	APOE	36/5.7	34/5.0	13	38	93.6	(K)AYKSELEEQLTPVAEETR(A)	3.9	2.383e^−4^
**1335**	P09525	Annexin A4	ANXA4	36/5.8	32/5.2	11	29	110.1	(K)AASGFNAmEDAQTLR(K)	3.2	0.002
**1343**	P67936	Tropomyosin alpha-4 chain	TPM4	28/4.7	32/4.2	5	19	88.0	(K)IQALQQQADEAEDR(A)	2.1	0.004
**1359**	P10768	S-formylglutathione hydrolase	ESD	31/6.5	32/5.8	3	16	63.0	(K)KAFSGYLGTDQSK(W)	2.4	0.002
**1386**	P62258	14-3-3 protein epsilon	YWHAE	29/4.6	31/4.1	9	33	83.7	(K)KVAGmDVELTVEER(N)	2.7	6.373e^−4^
**1570**	P02647	Apolipoprotein A-I	APOA1	31/5.6	28/4.5	10	36	101.4	(K)LLDNWDSVTSTFSK(L)	2.9	0.050
**1700**	P32119	Peroxiredoxin-2	PRDX2	22/9.1	25/5.0	4	17	57.9	(K)ATAVVDGAFKEVK(L)	3.9	9.910e^−5^
**1831**	P30086	Phosphatidylethanolamine-binding protein 1	PEBP1	21/7.0	24/6.1	4	23	53.6	(K)GNDISSGTVLSDYVGSGPPK(G)	2.0	0.025
**1904**	P02511	Alpha-crystallin B chain	CRYAB	20/6.8	23/5.9	4	20	42.9	(K)HFSPEELKVK(V)	2.8	0.006
**2046**	P00441	Superoxide dismutase [Cu-Zn]	SOD1	16/5.7	21/5.3	2	16	35.1	(R)HVGDLGNVTADK(D)	2.8	1.363e^−6^
**2276**	P62937	Peptidyl-prolyl cis-trans isomerase A	PPIA	18/7.7	19/6.0	5	36	66.0	(R)ALSTGEKGFGYK(G)	2.2	0.006
**2294**	P62937	Peptidyl-prolyl cis-trans isomerase A	PPIA	18/7.7	19/6.3	3	22	70.5	(R)VSFELFADKVPK(T)	2.1	0.020

Th: theoretical; Obs: observed; FV: fold variation.

**Table 3:** MS/MS data of protein spots up-regulated in WT FNA samples.

Spot no.	ID	Protein name	Gene name	Th	Obs	Matched peptides	Coverage (%)	Best ion score	Peptides identified	FV	p-value
				MW/pI	MW/pI						
**752**	P02768	Serum albumin	ALB	69/5.9	53/5.6	24	39	95.1	(K)AEFAEVSKLVTDLTK(V)	4.7	2.710e^−5^
**760**	P02768	Serum albumin	ALB	69/5.9	53/5.3	15	20	80.2	(K)LVAASQAALGL(-)	4.5	2.462e^−5^
**766**	P02768	Serum albumin	ALB	69/5.9	53/5.7	17	28	89.6	(K)LVAASQAALGL(-)	5.8	9.630e^−5^
**1057**	P02675	Fibrinogen beta chain	FGB	56/8.5	39/4.9	5	10	86.2	(K)LESDVSAQmEYcR(T)	2.1	2.447e^−4^
**1194**	P02768	Serum albumin	ALB	69/5.9	35/4.8	4	7	45.0	(R)FPKAEFAEVSK(L)	4.2	0.010
**1200**	P01876	Ig alpha-1 chain C region	IGHA1	38/6.1	35/5.7	6	20	80.0	(K)TFTcTAAYPESK(T)	3.0	1.289e^−4^
**1435**	P01857	Ig gamma-1 chain C region	IGHG1	36/8.5	31/5.9	6	23	59.3	(K)TTPPVLDSDGSFFLYSK(L)	6.5	6.774e^−4^
**1476**	P01834	Ig kappa chain C region	IGKC	12/5.6	30/6.6	3	48	46.5	(K)VDNALQSGNSQESVTEQDSK(D)	3.8	0.002
**1476**	P60174	Triosephosphate isomerase	TPI1	27/5.6	30/6.6	2	11	63.7	(K)QSLGELIGTLNAAK(V)	3.8	0.002
**1511**	P0CG05	Ig lambda-2 chain C regions	IGLC2	11/6.9	29/5.2	3	18	74.5	(R)SYScQVTHEGSTVEK(T)	5.4	1.341e^−5^
**1523**	P01834	Ig kappa chain C region	IGKC	12/5.6	29/5.7	4	54	65.0	(-)TVAAPSVFIFPPSDEQLK(S)	3.6	1.924e^−4^
**1523**	P0CG05	Ig lambda-2 chain C regions	IGLC2	11/6.9	29/5.7	3	48	58.2	(R)SYScQVTHEGSTVEK(T)	3.6	1.924e^−4^
**1525**	P01834	Ig kappa chain C region	IGKC	12/5.6	29/5.6	5	67	74.4	(K)VDNALQSGNSQESVTEQDSK(D)	2.7	0.002
**1525**	P0CG05	Ig lambda-2 chain C regions	IGLC2	11/6.9	29/5.6	2	30	60.8	(R)SYScQVTHEGSTVEK(T)	2.7	0.002
**1525**	P04792	Heat shock protein beta-1	HSPB1	23/6.0	29/5.6	2	13	60.7	(K)LATQSNEITIPVTFESR(A)	2.7	0.002
**1549**	P01834	Ig kappa chain C region	IGKC	12/5.6	29/5.7	2	36	54.9	(-)TVAAPSVFIFPPSDEQLK(S)	3.7	1.259e^−4^
**1559**	P01834	Ig kappa chain C region	IGKC	12/5.6	28/6.3	2	35	50.8	(K)VDNALQSGNSQESVTEQDSK(D)	3.3	8.485e^−4^
**1959**	P00738	Haptoglobin	HP	45/6.1	22/4.9	4	11	69.5	(R)TEGDGVYTLNDK(K)	2.3	4.768e^−4^
**2565**	P06702	Protein S100-A9	S100A9	13/5.7	13/5.3	4	32	43.8	(R)KDLQNFLK(K)	5.1	3.933e^−6^
**2627**	P01834	Ig kappa chain C region	IGKC	12/5.6	11/5.7	3	48	58.4	(K)VDNALQSGNSQESVTEQDSK(D)	5.5	0.004

Th: theoretical; Obs: observed; FV: fold variation.

### Validation of differentially expressed proteins in PA and WT FNA samples by WB analysis

**Figure 3:**
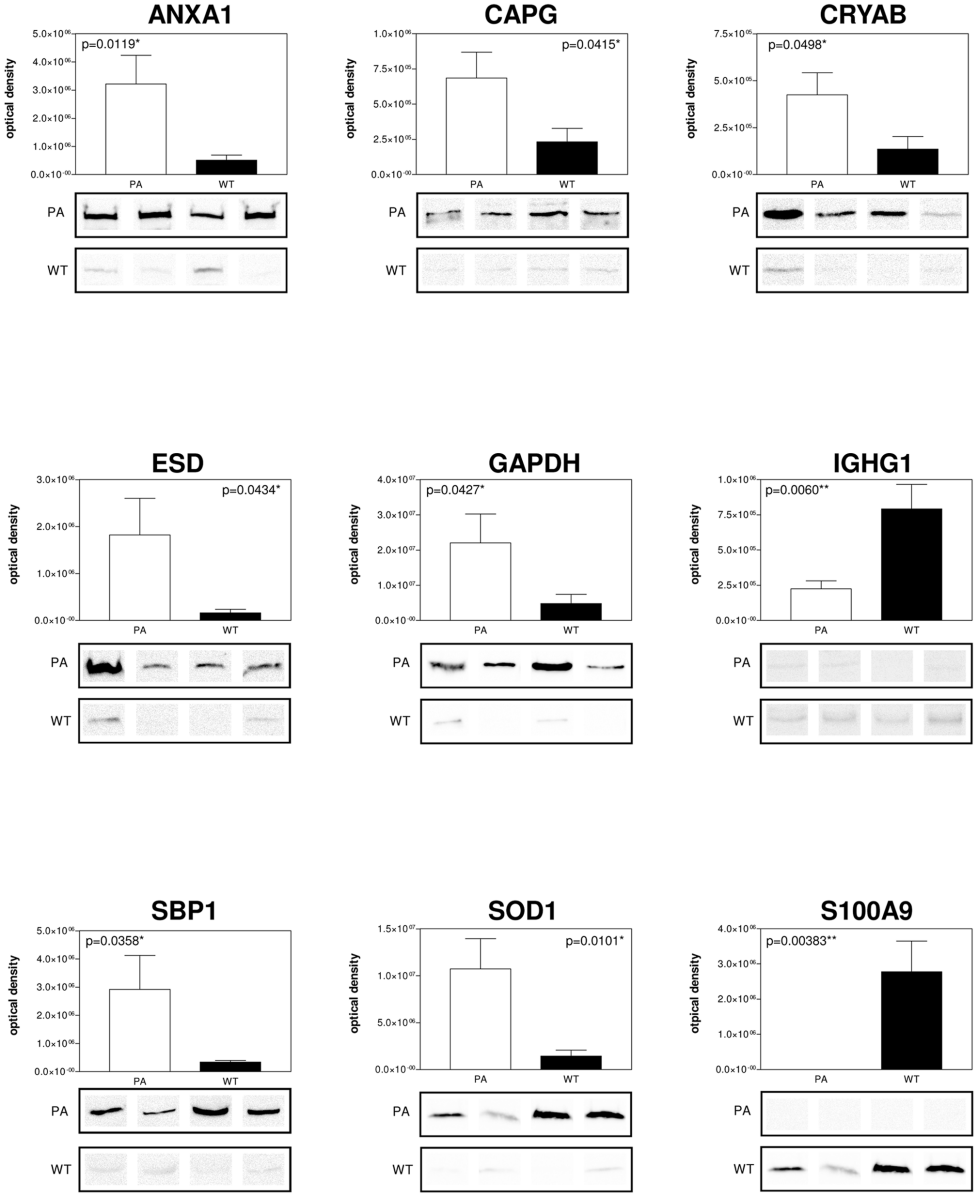
WB validation of selected differentially expressed proteins. ANXA1, CAPG, CRYAB, ESD, GAPDH, IGHG1, SBP1, SOD, S100A9 different expression by immunoblot analysis. Conventional SDS gels were run with protein extracts from PA and WT FNA samples from all samples using 12% resolving capacity. Thirty micrograms of total proteins were loaded into each lane for CAPG, CRYAB, ESD, GAPDH, SBP1, SOD, S100A9 WB, while 10 µg were loaded for ANXA1 and IGHG1 WB. Proteins were transferred onto nitrocellulose membranes and incubated with specific antibodies against the target proteins. Four lanes for PA and WT are shown for each antibody and densitometry of the blots is shown on the top. Statistically significant differences were determined by the Student t test and the p values are indicated.

WB analysis, using specific polyclonal antibodies, was used to validate the expression changes of some of the proteins identified by 2DE. A subset of 9 candidate proteins was selected for validation by immunoassays; in particular, the different expression of ANXA1, CAPG, CRYAB, ESD, GAPDH, S100A9, SOD1, SBP1 and IGHG1 was confirmed. All PA and WT FNA samples were analysed. For each tested protein, the optical density of specific immunoreactive band was determined and the resulting mean values ± SEM were compared (Fig. 3). From the comparison of PA and WT FNA samples, an increase of expression both of ANXA1 (p = 0.0119), CAPG (p = 0.0415), CRYAB (p = 0.0498), ESD (p = 0.0434), GAPDH (p = 0.0427), SOD (p = 0.0101), SBP1 (p = 0.0358) and of S100A9 (p = 0.00383), IGHG1 (p = 0.0060) was confirmed in PA and WT samples, respectively.

### Network construction for biological processes

**Figure 4:**
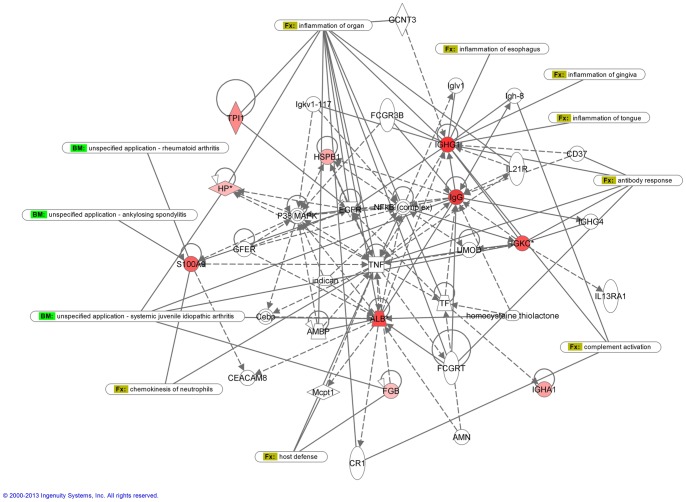
IPA network analysis of WT up-regulated proteins. The network is associated with immunological and inflammatory disease. Solid lines correspond to direct protein-to-protein interactions/regulations, dashed lines correspond to indirect interactions/regulations.

**Figure 5:**
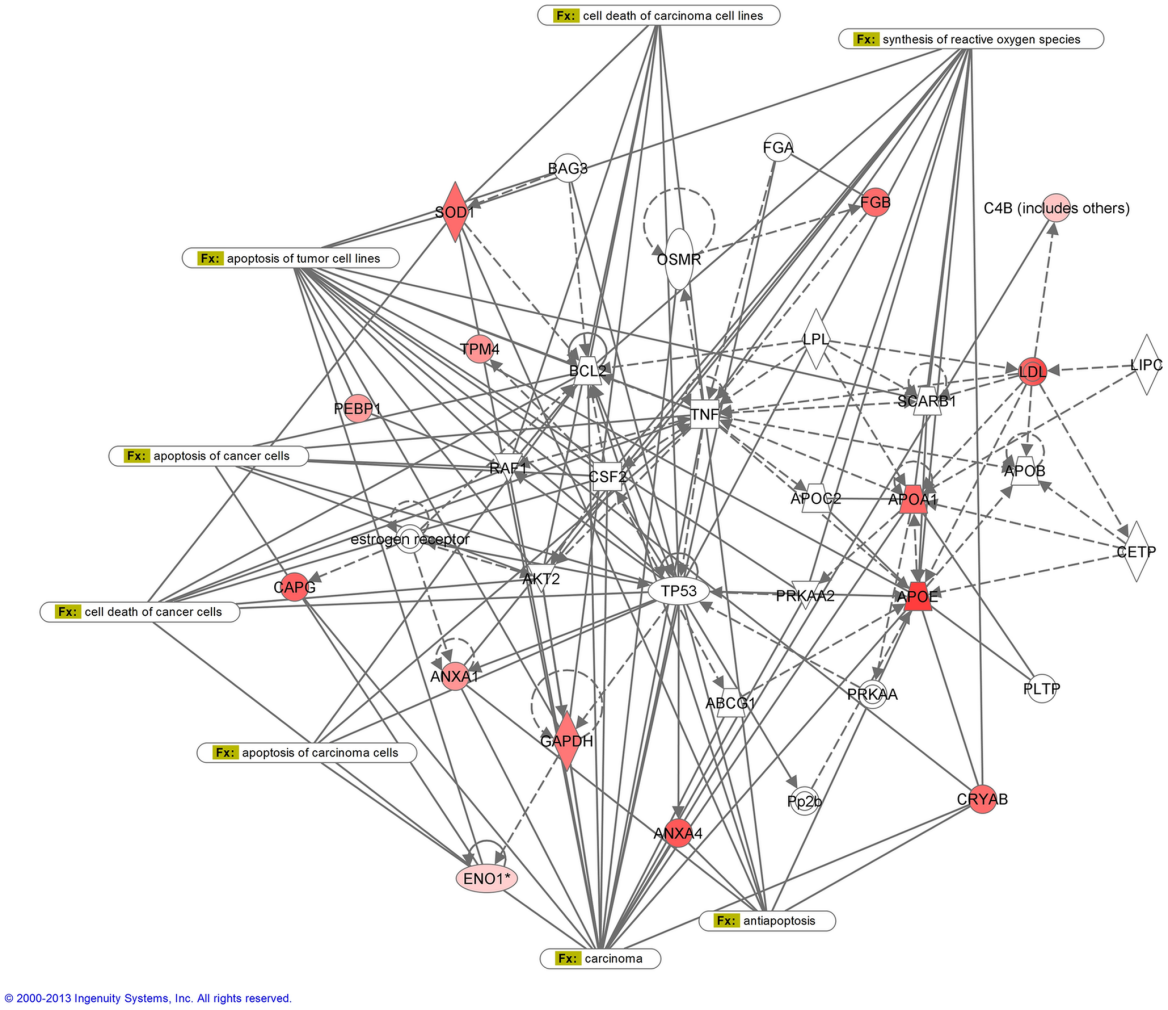
IPA network analysis of PA up-regulated proteins. The network is associated with cancer disease. Solid lines correspond to direct protein-to-protein interactions/regulations, dashed lines correspond to indirect interactions/regulations.

The assignment of biological processes and the subsequent construction of networks were done using the Ingenuity software. All the proteins that had a >2.0-fold statistically significant change in expression between PA and WT FNA were included in the analysis. Each identified protein was converted to its gene and mapped to its corresponding gene object in the IPA knowledge base. Two networks with a score value of 27 (WT network) and 29 (PA network) were generated (Fig. 4 and 5). Genes or gene products are represented as nodes, and the biological relationship between two nodes is represented as an edge (line). IPA was utilized to retrieve the known functions of each protein. WT network with highest score shows 35 proteins containing 10 of the up-regulated proteins. This network is associated with immunological and inflammatory disease. The diseases associated to this analysis are immunological (p-value 1.42E-2 – 4.93E-2) and inflammatory diseases (p-value 1.42E-2 – 4.93E-2). PA network shows 35 proteins, 12 of them found to be up-regulated in PA samples, that are associated with cancer disease (p-value 7.35E-6 – 4.56E-2).

## Discussion

Among parotid neoplasms, misdiagnosis can occur, leading to unnecessary surgical intervention. For instance, from literature reports, it emerges that even if it is still a matter of debate, it can be stated that the development of WT is related to the epithelial cells of the heterotopic salivary gland in the intraparotid lymph nodes [19]. A mixture of oncocytic epithelial fragments and lymphocytes in a proteinaceous background is a typical FNA cytologic finding. Despite these typical cytological features, however, the variegated morphology may lead to an erroneous cytopathological interpretation and can make it difficult to differentiate from benign and malignant neoplasms. At present, no genetic or protein biomarkers have been advanced to enhance the accuracy of parotid tumour cytological examination. In previous studies we demonstrated the applicability of FNA proteomics to examine thyroid tumours [13], [14]. Now, in this study, for the first time, we performed a proteomic comparative analysis of WT and PA parotid FNA fluids in order to define potential protein biomarkers able to improve pre-operative diagnosis. Thirty-four differentially expressed protein spots, with a fold variation major than 2, were found. Among them, 16 resulted up-regulated in WT with respect to PA, while a down regulation was observed for the residual 18 proteins. Identification by mass spectrometry led to the identification of 26 different proteins. IPA analysis, performed on 16 up-regulated WT proteins, associated with “Humoral Immune Response, Protein Synthesis, Connective Tissue Disorders” network with the Top Bio Functions related to connective tissue disorders, immunological and inflammatory diseases. As shown by IPA network, changes of S100A9 protein expression levels are known in literature to be associated with autoimmune systemic inflammatory diseases such as rheumatoid arthritis [20] and diffuse systemic sclerosis [21], [22] while an increase of IGKC and IGHA1 proteins was found in saliva belonging to non-Hodgkin's lymphoma patient in comparison with primary Sjögren syndrome [23], directly correlated to the lymphoproliferative process. This observation agrees with previous studies showing that WT is characterized by a strong immunologic reaction, indeed Daguci and co-workers have reported the presence of B-cells (CD20), NK (CD56) and T (CD3), including helper subtypes (CD4) and suppressor (CD8) in the WT's stroma, something similar to that of normal or reactive lymph nodes. Moreover, there is a strong positivity towards markers of lymphocyte proliferation (CD20cy and CD45RO) [24]. These histological and cytoarchitectural aspects are in line with the proteomic profile emerged in our findings where many proteins related to immune response were found. In particular, we described the overexpression of IGHG1, IGKC, IGHA1 and IgG chains probably directly or indirectly induced from some inflammatory mediators such as tumour necrosis factor (TNF). Conversely, the IPA network obtained from the analysis of PA up-regulated proteins, associated to Lipid Metabolism, Small Molecules Biochemistry, Molecular Transport, with the Top Bio functions and diseases related to cell death and survival, free radical scavenging, cancer and inflammatory disease. Fourteen of these proteins were included in the network and are known to be candidate biomarkers in several types of tumours, where are involved in proliferation, invasiveness and apoptotic processes and then synthesis of reactive oxygen species.

As far as CapG is concerned, a 3.0 fold variation in 2D gels analysis and a 2.4 fold variation in WB analysis in PA *vs* WT samples was observed. CapG was originally identified as the protein that binds to the barbed end of acting filaments [25]. It was found to be overexpressed in lung cancer [26], cholangiocarcinoma [27], colorectal cancer [28], breast cancer [29], ovarian cancer [30], oral cancer [31], pancreatic cancer [32] and in nasopharyngeal carcinoma [33]. CapG appears to play an important role in the process of metastasis by promoting the invasiveness of tumour cells as reported in hepatocellular carcinoma. Kimura [34] compared hepatocellular carcinoma with and without vascular invasion and found that those with vascular invasion showed markedly up-regulated expression of CapG. Moreover, silencing of CapG reduced tumour invasion without affecting the proliferation of the HCC cells.

With regards to CRYAB, different and sometimes opposite roles are described in literature. Our results suggested a strong increase of CRYAB in PA *vs* WT confirmed also by WB analysis. CRYAB is a member of the small heat shock protein (sHSP) family, is expressed in various tissues where it acts as a molecular chaperone, maintains protein conformation and prevents protein aggregation. This role is described in the cells at physiological levels of CRYAB. In contrast, when overexpressed, it appears to prevent apoptosis through the interaction with the common apoptotic protein pathways [35].

Besides CRYAB, annexins (ANXA1 and ANXA4) and Apolipoprotein E (APOE) are known to have antiapoptotic function acting by different mechanisms of action. In particular, annexins play important roles in different tumours such as hepatocellular carcinoma, adenocarcinoma of the breast and prostate and head and neck squamous cell carcinoma, where alone/or synergistically regulate apoptosis, carcinogenesis, migration and invasion of tumour cells [36]. In our network, all above mentioned proteins directly interact with TP53 protein. Prediction of the upstream and downstream effects of activation and inhibition on other network proteins, suggests that a simulation of TP53 activation agrees with the observed increase of ANXA1, ANXA4, APOE. These observations are according with an imbalance of apoptotic response as already observed in other adenomas [37].

Overall, our results shed new light on the potential usefulness of a proteomic approach to study parotid tumours, and most importantly they highlight as upregulated proteins from WT and PA generate different networks respectively, with the first correlated to inflammatory and immunity events and the second to early tumorigenesis process such as apoptosis, cellular growth and stress response. These different features might be useful in the differential diagnosis of benign parotid tumours. In fact the identification of new markers may improve the clinical work allowing a more specific diagnosis and consequently, an appropriate clinical treatment of patients with salivary parotid lesions. Last but not least these markers could overcome the limit of cytology and could lead to an advantageous reduction of surgical interventions.

## References

[1] Eveson JW, Cawson RA (1985) Tumours of the minor (oropharyngeal) salivary glands: a demographic study of 336 cases. J Oral Pathol 14: 500–509. 2991488 10.1111/j.1600-0714.1985.tb00522.x

[2] Heller KS, Dubner S, Chess Q, Attie JN (1992) Value of fine needle aspiration biopsy of salivary gland masses in clinical decision-making. Am J Surg 164: 667–670. 1463121 10.1016/s0002-9610(05)80731-7

[3] Fakhry N, Antonini F, Michel J, Penicaud M, Mancini J, et al (2012) Fine-needle aspiration cytology in the management of parotid masses: evaluation of 249 patients. Eur Ann Otorhinolaryngol Head Neck Dis 129: 131–135. 22626640 10.1016/j.anorl.2011.10.008

[4] Tryggvason G, Gailey MP, Hulstein SL, Karnell LH, Hoffman HT, et al (2013) Accuracy of fine-needle aspiration and imaging in the preoperative workup of salivary gland mass lesions treated surgically. Laryngoscope 123: 158–163. 22991236 10.1002/lary.23613

[5] Nagel H, Hotze HJ, Laskawi R, Chilla R, Droese M (1999) Cytologic diagnosis of adenoid cystic carcinoma of salivary glands. Diagn Cytopathol 20: 358–366. 10352908 10.1002/(sici)1097-0339(199906)20:6<358::aid-dc6>3.0.co;2-x

[6] Stewart CJ, MacKenzie K, McGarry GW, Mowat A (2000) Fine-needle aspiration cytology of salivary gland: a review of 341 cases. Diagn Cytopathol 22: 139–146. 10679992 10.1002/(sici)1097-0339(20000301)22:3<139::aid-dc2>3.0.co;2-a

[7] Yaranal PJ, Umashankar T (2013) Squamous Cell Carcinoma Arising in Warthin's Tumour: A Case Report. J Clin Diagn Res 7: 163–165. 23449505 10.7860/JCDR/2012/4683.2697 PMC3576778

[8] Viguer JM, Vicandi B, Jiménez-Heffernan JA, López-Ferrer P, González-Peramato P, et al (2010) Role of fine needle aspiration cytology in the diagnosis and management of Warthin's tumour of the salivary glands. Cytopathology. 21: 164–169. 10.1111/j.1365-2303.2009.00667.x 19744189

[9] Tripathi SC, Matta A, Kaur J, Grigull J, Chauhan SS, et al (2010) Nuclear S100A7 is associated with poor prognosis in head and neck cancer. PLoS One 5: e11939. 20689826 10.1371/journal.pone.0011939 PMC2914786

[10] Tripathi SC, Matta A, Kaur J, Grigull J, Chauhan SS, et al (2011) Overexpression of prothymosin alpha predicts poor disease outcome in head and neck cancer. PLoS One 6: e19213. 21573209 10.1371/journal.pone.0019213 PMC3088661

[11] Ralhan R, Masui O, Desouza LV, Matta A, Macha M, et al (2011) Identification of proteins secreted by head and neck cancer cell lines using LC-MS/MS: Strategy for discovery of candidate serological biomarkers. Proteomics 11: 2363–2376. 21598386 10.1002/pmic.201000186

[12] Gonzalez-Begne M, Lu B, Han X, Hagen FK, Hand AR, et al (2009) Proteomic analysis of human parotid gland exosomes by multidimensional protein identification technology (MudPIT). J Proteome Res 8: 1304–1314. 19199708 10.1021/pr800658c PMC2693447

[13] Giusti L, Iacconi P, Ciregia F, Giannaccini G, Basolo F, et al (2007) Proteomic analysis of human thyroid fine needle aspiration fluid. J Endocrinol Invest 30: 865–869. 18075290 10.1007/BF03349229

[14] Giusti L, Iacconi P, Ciregia F, Giannaccini G, Donatini GL, et al (2008) Fine-needle aspiration of thyroid nodules: proteomic analysis to identify cancer biomarkers. J Proteome Res 7: 4079–4088. 18665625 10.1021/pr8000404

[15] Eveson JW, Auclair P, Gnepp DR (2005) Tumors of the salivary glands. Barnes L, Eveson J, Reichart P, Sidransky D (Eds.), Pathology and Genetics of Head and Neck Tumors. World Health Organization Classification of Tumors, IARC Press, Lyon, 209–281.

[16] Giusti L, Cetani F, Ciregia F, Da Valle Y, Donadio E, et al (2011) A proteomic approach to study parathyroid glands. Mol Biosyst 7: 687–699. 21180715 10.1039/c0mb00191k

[17] Aude-Garcia C, Collin-Faure V, Luche S, Rabilloud T (2011) Improvements and simplifications in in-gel fluorescent detection of proteins using ruthenium II tris-(bathophenanthroline disulfonate): the poor man's fluorescent detection method. Proteomics 11: 324–328. 21204259 10.1002/pmic.201000370

[18] Soggiu A, Piras C, Bonizzi L, Hussein HA, Pisanu S, et al (2012) A discovery-phase urine proteomics investigation in type 1 diabetes. Acta Diabetol 49: 453–464. 22678621 10.1007/s00592-012-0407-0

[19] Teymoortash A, Werner JA, Moll R (2011) Is Warthin's tumour of the parotid gland a lymph node disease? Histopathology 59: 143–145. 21771031 10.1111/j.1365-2559.2011.03891.x

[20] Giusti L, Baldini C, Ciregia F, Giannaccini G, Giacomelli C, et al (2010) Is GRP78/BiP a potential salivary biomarker in patients with rheumatoid arthritis? Proteomics Clin Appl 4: 315–324. 21137052 10.1002/prca.200900082

[21] Giusti L, Baldini C, Bazzichi L, Ciregia F, Tonazzini I, et al (2007) Proteome analysis of whole saliva: a new tool for rheumatic diseases--the example of Sjögren's syndrome. Proteomics 7: 1634–1643. 17436266 10.1002/pmic.200600783

[22] Giusti L, Bazzichi L, Baldini C, Ciregia F, Mascia G, et al (2007) Specific proteins identified in whole saliva from patients with diffuse systemic sclerosis. J Rheumatol 34: 2063–2069. 17722226

[23] Baldini C, Giusti L, Ciregia F, Da Valle Y, Giacomelli C, et al (2011) Correspondence between salivary proteomic pattern and clinical course in primary Sjögren syndrome and non-Hodgkin's lymphoma: a case report. J Transl Med 9: 188. 22047044 10.1186/1479-5876-9-188 PMC3223154

[24] Dăguci L, Simionescu C, Stepan A, Munteanu C, Dăguci C, et al (2011) Warthin tumor–morphological study of the stromal compartment. Rom J Morphol Embryol 52: 1319–1323. 22203940

[25] Casella JF, Maack DJ, Lin S (1986) Purification and initial characterization of a protein from skeletal muscle that caps the barbed ends of actin filaments. J Biol Chem 261: 10915–10921. 3733738

[26] Ha ES, Choi S, In KH, Lee SH, Lee EJ, et al (2013) Identification of proteins expressed differently among surgically resected stage I lung adenocarcinomas. Clin Biochem 46: 369–377. 23200884 10.1016/j.clinbiochem.2012.11.014

[27] Morofuji N, Ojima H, Onaya H, Okusaka T, Shimada K, et al (2012) Macrophage-capping protein as a tissue biomarker for prediction of response to gemcitabine treatment and prognosis in cholangiocarcinoma. J Proteomics 75: 1577–1589. 22155129 10.1016/j.jprot.2011.11.030

[28] Wu JH, Tian XY, Hao CY (2011) The significance of a group of molecular markers and clinicopathological factors in identifying colorectal liver metastasis. Hepatogastroenterology 58: 1182–1188. 21937374 10.5754/hge11380

[29] Renz M, Betz B, Niederacher D, Bender HG, Langowski J (2008) Invasive breast cancer cells exhibit increased mobility of the actin binding protein CapG. Int J Cancer 122: 1476–1482. 18059028 10.1002/ijc.23215

[30] Partheen K, Levan K, Osterberg L, Claesson I, Fallenius G, et al (2008) Four potential biomarkers as prognostic factors in stage III serous ovarian adenocarcinomas. Int J Cancer 23: 2130–2137. 10.1002/ijc.23758 18709641

[31] Nomura H, Uzawa K, Ishigami T, Kouzu Y, Koike H, et al (2008) Clinical significance of gelsolin-like actin-capping protein expression in oral carcinogenesis: an immunohistochemical study of premalignant and malignant lesions of the oral cavity. BMC Cancer 8: 39. 18237446 10.1186/1471-2407-8-39 PMC2263057

[32] Thompson CC, Ashcroft FJ, Patel S, Saraga G, Vimalachandran D, et al (2007) Pancreatic cancer cells overexpress gelsolin family-capping proteins, which contribute to their cell motility. Gut 56: 95–106. 16847067 10.1136/gut.2005.083691 PMC1856675

[33] Li MX, Xiao ZQ, Chen YH, Peng F, Li C, et al (2010) Proteomic analysis of the stroma-related proteins in nasopharyngeal carcinoma and normal nasopharyngeal epithelial tissues. Med Oncol 27: 134–144. 19242827 10.1007/s12032-009-9184-1

[34] Kimura K, Ojima H, Kubota D, Sakumoto M, Nakamura Y, et al (2013) Proteomic identification of the macrophage-capping protein as a protein contributing to the malignant features of hepatocellular carcinoma. J Proteomics 78: 362–373. 23085225 10.1016/j.jprot.2012.10.004

[35] Volkmann J, Reuning U, Rudelius M, Häfner N, Schuster T, et al.. (2012) High expression of crystallin αB represents an independent molecular marker for unfavourable ovarian cancer patient outcome and impairs TRAIL- and cisplatin-induced apoptosis in human ovarian cancer cells. Int J Cancer doi: 10.1002/ijc.27975. 10.1002/ijc.27975 23225306

[36] Mussunoor S, Murray GI (2008) The role of annexins in tumour development and progression. J Pathol 216: 131–140. 18698663 10.1002/path.2400

[37] Giusti L, Cetani F, Ciregia F, Da Valle Y, Donadio E, et al (2011) A proteomic approach to study parathyroid glands. Mol Biosyst 7: 687–699. 21180715 10.1039/c0mb00191k

